# The Effects of Silymarin on Calcium Chloride-Induced Arrhythmia in Male Rat

**DOI:** 10.1155/2024/6720138

**Published:** 2024-08-31

**Authors:** Fereshteh Toghroli, Mohammad Foad Noorbakhsh, Javad Sajedianfard

**Affiliations:** Department of Basic Sciences School of Veterinary Medicine Shiraz University, Shiraz, Iran

## Abstract

Antioxidants play an important role in protecting cardiac arrhythmias. Silymarin, strong antioxidant, is effective in reducing the complications caused by arrhythmias. This study was conducted to determine the effect of silymarin on the prevention and treatment of calcium chloride-induced arrhythmia. In total, 48 male rats were randomly divided into six groups: the first control group for acute administration received intravenous injection of 0.2 mL of dimethylsulfoxide, a cosolvent, immediately after induction of arrhythmia; the second control group for chronic administration, daily gavage of dimethylsulfoxide for 2 weeks before induction of arrhythmia; acute silymarin group, 100 mg/kg intravenous, immediately after the occurrence of arrhythmia; chronic silymarin group, daily gavage of 50 mg/kg for 2 weeks before induction of arrhythmia; amiodarone standard treatment, 5 mg/kg intravenous, immediately after induction of arrhythmia; and quinidine standard treatment, 10 mg/kg intravenous, immediately after induction of arrhythmia. Calcium chloride (140 mg/kg, i.v.) was used to induce arrhythmia. Electrocardiogram was recorded and monitored by PowerLab™ system. The incidence rates of premature ventricular beat (PVB), ventricular tachycardia (VT), and ventricular fibrillation (VF) were calculated. The antiarrhythmic effect of silymarin was observed with a significant decrease in the incidence of premature ventricular beat (22.56 ± 1.04%, *P* < 0.001), ventricular tachycardia (34.150 ± 1.59%, *P* < 0.001), and ventricular fibrillation (24.31 ± 1.02%, *P* < 0.001) compared with the control group (100%). These effects were comparable to antiarrhythmic drugs such as quinidine (29.23% ± 1.24%, 52.23% ± 1.13%, 66.31% ± 1.81%) and amiodarone (22.91% ± .72%, 41.09% ± 1.66%, 61.59% ± 1.11%). Silymarin exerts a potent antioxidant effect, thereby mitigating the risk of VT, VF, and PVC.

## 1. Introduction

Cardiovascular diseases are among the important causes of death in the world [1, 2]. The mortality rate attributable to cardiovascular diseases in the world is estimated to be more than 24 million people by 2030 [1]. Tachyarrhythmia and ventricular fibrillation (VF) are the most important causes of sudden cardiac death [3] Atrial fibrillation is associated with an increase in thromboembolism, which can increase mortality and impose substantial health costs [4, 5, 6, 7, 8].

Common treatments for arrhythmia include antiarrhythmic drugs, implantable cardiac defibrillators, and cardiac ablation. There are limitations in the use of these treatments. For example, long-term use of class IC antiarrhythmic drugs has increased cardiac mortality in heart attack patients [9]. The reason for the limitation of many common treatments is that they do not affect the pathophysiology of arrhythmia. Treatment based on the pathology that caused the abnormal state of ion flow, focal activity, and reentry will also be more successful. Oxidative stress is an example of upstream therapeutic targets. Most of the clinical risk factors of atrial fibrillation (AF), such as hypertension, age, and cardiac surgery, are disorders [10] associated with oxidative stress [11]. Serum markers of oxidative stress are increased in patients with AF [10]. Cardiomyopathy is associated with oxidative stress, which significantly increases the incidence of ventricular tachycardia (VT) and VF [12]. Antioxidants play an important role in preventing and treating cardiac arrhythmias by combating the effects of oxidative stress. Antioxidants, such as vitamins C and E, glutathione, superoxide dismutase, catalase, allopurinol, and mito-tempo, can directly react with ROS or indirectly inhibit free radical-generating enzymes and activate intracellular antioxidant enzymes. While some studies have demonstrated the potential benefits of antioxidant therapy in preventing and treating arrhythmias, especially in the case of atrial fibrillation, other studies have failed to show a clear therapeutic effect. In conclusion, while the role of oxidative stress in the pathogenesis of arrhythmias is well-established, the clinical utility of antioxidant therapy in arrhythmia management remains a subject of ongoing research and debate [13].

Currently, due to the occurrence of complications with chemical drugs, much attention has been paid to the screening of herbal drugs used in traditional medicine. According to the World Health Organization, 80% of the world's population uses herbal medicines [14]. The extract of *Silybum marianum* contains various flavonolignans, which are called silymarin [15]. Silymarin is a mixture of four flavonolignan isomers including silybin, iso-silybin, silydianin, and silychristin. Silybin is the most effective biological substance of silymarin [16]. Silymarin has been shown to be a protective agent for neurons and hepatic, kidney, and heart cells due to its antioxidant and tissue-regenerating effects [17, 18, 19, 20]. Silymarin exerts its antioxidant effects in various ways including scavengering free radicals [21, 22]. Preventing the formation of free radicals by inhibiting enzymes or maintaining the electron transport chain in mitochondria under stress conditions [23] and participating in maintaining the optimal state of regeneration by activating enzymatic and nonenzymatic antioxidants. Oxidants are created through transcription factors such as Nrf2 and NF-*κ*B [24, 25] activation of vitagens responsible for the production of protective molecules including heat-shock protein (HSP) and thioredoxin and providing more protection in stress conditions [26, 27]. As a result of its antioxidant effects, silymarin has been able to prevent cardiovascular diseases [28]. In the study of Radmanesh et al. [28], it has been shown that calcium chloride (CaCl_2_), while causing arrhythmia in rats, causes an increase in malonyl aldehyde (MDA) and a decrease in antioxidant factors such as superoxide dismutase (SOD) and catalase [29].

Oxidative stress, characterized by an imbalance between reactive oxygen species (ROS) and antioxidant defenses, is a key factor in the development of various types of arrhythmias, including atrial fibrillation (AF), ventricular tachycardia (VT), and ventricular fibrillation (VF). On the other hand, antioxidants have been shown to have antiarrhythmic effects in various experimental studies. Based on the review literature, silymarin's effect on calcium chloride-induced arrhythmia has not been studied. Considering that silymarin is a strong antioxidant; in the present study, the effect of silymarin in the prevention and treatment of arrhythmia caused by CaCl_2_ has been investigated.

## 2. Materials and Methods

### 2.1. Animals

Male Sprague–Dawley rats weighing between 300 and 350 g were used in this study. Every three rats were placed in a cage. The tested animals were kept in the animal house with 12 : 12 hr of darkness and light at a temperature of 22 ± 2°C and 50% humidity. Animals had free access to pelleted food and sanitary water. All experiments were performed according to the instructions of the ethics committee of Shiraz University (98GCU1M1293).

### 2.2. Study Design

Forty-eight rats were randomly divided into the following six groups:

1. Group 1: Negative control 1 (for acute administration), received intravenous (i.v.) injection of 0.2 mL of dimethylsulfoxide (DMSO, as a drug carrier) immediately after induction of arrhythmia.
2. Group 2: Negative control 2 (for chronic administration), received daily gavage of DMSO (as a drug carrier) for 2 weeks before induction of arrhythmia.
3. Group 3: Treatment 1 (silymarin A), received i.v. injection of 100 mg/kg silymarin (Sigma, USA) immediately after the occurrence of arrhythmia. The silymarin used was a yellow powder and DMSO and distilled water were used to increase the volume.
4. Group 4: Treatment 2 (silymarin), received 50 mg/kg of silymarin (Sigma, USA) orally for 2 weeks before induction of arrhythmia.
5. Group 5: Amiodarone (Sigma, USA) standard treatment, received 5 mg/kg amiodarone i.v. immediately after occurrence of arrhythmia.
6. Group 6: Quinidine (Sigma, USA) standard treatment, received 10 mg/kg quinidine i.v. immediately after occurrence of arrhythmia. In this group, sodium chloride was used to increase the volume.

### 2.3. Arrhythmia Record

The animals were anesthetized by intraperitoneal (i.p.) injection of 50 mg/kg pentobarbital sodium (Sigma, USA). After the induction of anesthesia, the animals were placed on their back to record the electrocardiogram. For better ECG recording, lead II needle electrodes were attached subcutaneously to both forelimbs and the left hindlimb of the animal; cardiac rhythm was monitored by the Powerlab™ system and bioamplifier (ADInstrument, Australia).

To prevent possible blood coagulation and to ensure that the injection path remains open, 0.2 mL heparin was injected for each rat before the induction of arrhythmia. To induce arrhythmia, a polyethylene catheter was placed in the tail vein, and 140 mg/kg of 2.5% CaCl_2_ solution was injected. The test solution was also injected into each group through a catheter placed in another tail vein. ECG was recorded from 15 min before induction of arrhythmia up to 45 min after the occurrence of arrhythmia. The incidence rates of premature ventricular beats (PVB), ventricular tachycardia (VT), and ventricular fibrillation (VF) were calculated at 1- and 3-min after induction of arrhythmia and presented as a percentage compared to the control group. The heart rate was also recorded during this period.

### 2.4. Heart Tissue Extraction

For this purpose, 1 g of heart tissue was separated and transferred to a test tube after weighing. Five milliliters of phosphate buffer was then dropped on the hepatic tissue. The mixture of tissue and phosphate buffer was well homogenized using a homogenizer. The resulting solution was centrifuged (at 2,500 rpm for 5–10 min). The supernatant was then collected and used to measure the desired parameters.

### 2.5. Measuring the Total Antioxidant Capacity (TAC) and Malondialdehyde (MDA) in the Heart

A commercial kit (Zell Bio GmbH kit, Germany) was used to determine the TAC level. The color product of the chromogenic substrate (tetramethylbenzidine) emerged at the ending phase. The difference in color was calculated calorimetrically using a spectrophotometer (Jenway 6300 Spectrophotometer, UK) at 450 nm and represented as mmol/L. This method can determine TAC with 0.1 mM sensitivity (100 *μ*mol/L). The intra- and inter-assay CVs were below 3.4% and 4.2%, respectively. An assay kit purchased from ZellBio GmbH (Germany) was used to measure MDA (*μ*mol/L; Cat. no. ZB-MDA96A). In this kit, MDA is measured based on its reaction with thiobarbituric acid in an acidic condition and high temperature. The color complex was measured colorimetrically at 535 nm. The assay kit sensitivity was 0.1 *μ*M (interassay CV: 5.8%) for MDA.

### 2.6. Statistical Methods

Graphpad® Prism™ 8 software was used for data analysis. Data are presented as mean ± standard error of the mean. Two-way ANOVA and Tukey's HSD as post hoc tests were used to analyze the data. A *P* value < 0.05 was considered statistically significant. The effect size (Cohen's d) was calculated and reported for all pairwise comparisons.

## 3. Results

### 3.1. Based on the Result of the Kolmogorov–Smirnov Test, the Obtained Data Had a Normal Distribution

#### 3.1.1. The Effect of Chronic and Acute Administration of Silymarin on CaCl_2_-Induced Arrhythmia 1 Min after the Induction of Arrhythmia in Comparison with Amiodarone

**Figure 1:**
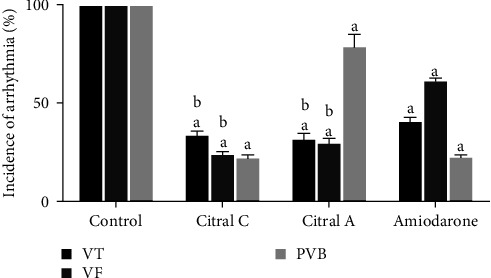
The effect of chronic (50 mg/kg administered i.p. over 2 weeks, silymarin) and acute (100 mg/kg administered i.v. immediately after induction of arrhythmia, silymarin A) administration of silymarin on arrhythmia caused by CaCl_2_ 1 min after the induction of arrhythmia in comparison with amiodarone (5 mg/kg). The information in the control group was considered 100% and was compared with other groups. PVB, premature ventricular beat; VT, ventricular tachycardia; VF, ventricular fibrillation. ^a^*P*  < 0.001 against the control group; ^b^*P*  < 0.001, against amiodarone.

The incidence of VT, VF, and PVB occurred in all three studied groups were compared with the control group 1 min after induction of arrhythmia. The incidence rates of VT (*P* < 0.001, *d* = 0.976) and VF (*P* < 0.001, *d* = 0.986) recorded in all three studied groups were significantly lower than those in the control group; the rates in the chronic (*P* < 0.001, *d* = 0.936) and acute (*P* < 0.001, *d* = 0.964) silymarin groups were significantly lower than that in the amiodarone group. The incidence of PVB had similar changes except that the reduction in the incidence of PVB in the chronic and acute silymarin groups did not significantly decrease compared to the amiodarone group. The incidence of various types of arrhythmias in the acute silymarin group decreased compared to the chronic silymarin group, but it was not significant (Figure 1).

#### 3.1.2. The Effect of Chronic and Acute Administration of Silymarin on CaCl_2_-Induced Arrhythmia after 1 Min of Induction of Arrhythmia Compared with Quinidine

**Figure 2:**
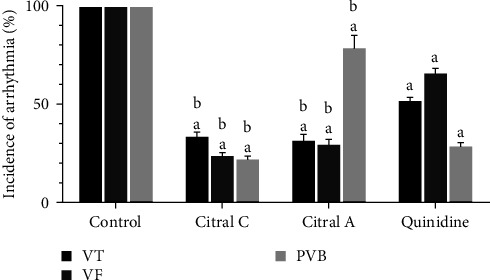
The effect of chronic (50 mg/kg i.p. administration over 2 weeks, silymarin) and acute (100 mg/kg i.v. administration immediately after induction of arrhythmia, silymarin A) administration of silymarin on CaCl_2_-induced arrhythmia 1 min after the induction of arrhythmia in comparison with quinidine (10 mg/kg). The information in the control group was considered 100% and was compared with other groups. PVB, premature ventricular beat; VT, ventricular tachycardia; and VF, ventricular fibrillation. ^a^*P*  < 0.001 against the control group; ^b^*P*  < 0.001 against quinidine.

The incidence rates of VT, VF, PVB 1 min after induction of arrhythmia recorded in all three groups significantly decreased compared to those in the control group (Figure 2). In the silymarin group, where the drug was injected immediately after induction of arrhythmia, the incidence rates of VT (*P* < 0.001, *d* = 0.976), VF (*P* < 0.001, *d* = 0.986), and PVB (*P* < 0.001, *d* = 0.953) significantly decreased in all studied groups compared to the control group. The incidence of arrhythmias in the silymarin treatment groups was significantly reduced compared to the standard quinidine group (*P* < 0.001, *d* = 0.909). Although the incidence of arrhythmias in the silymarin acute treatment group decreased compared to the chronic treatment group, the difference was not significant (*P*=0.99).

#### 3.1.3. The Effect of Chronic and Acute Administration of Silymarin on CaCl_2_-Induced Arrhythmia 3 Min after the Induction of Arrhythmia in Comparison with Amiodarone

**Figure 3:**
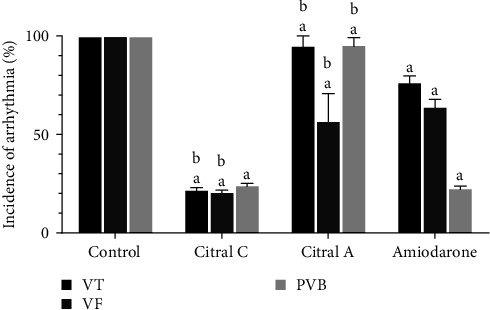
The effect of chronic (50 mg/kg i.p. administration over 2 weeks, silymarin) and acute (100 mg/kg i.v. administration immediately after induction of arrhythmia, silymarin A) administration of silymarin on CaCl_2_-induced arrhythmia 3 min after induction of arrhythmia in comparison with amiodarone (5 mg/kg). The information in the control group was considered 100% and was compared with other groups. PVB, premature ventricular beat; VT, ventricular tachycardia; and VF, ventricular fibrillation. ^a^*P*  < 0.001 against the control group; ^b^*P*  < 0.001 against amiodarone.

Chronic and acute administration of silymarin significantly (*P* < 0.001, *d* = 0.965) decreased the incidence of arrhythmias 3 min after induction of arrhythmia compared to the control group. In the chronic silymarin group, the decrease in the incidence of VT (*P* < 0.001, *d* = 0.911) and VF (*P* < 0.001, *d* = 0.737) was significantly less than that of amiodarone group. In the acute silymarin group, the incidence of all three types of arrhythmias decreased significantly (*P* < 0.001, *d* = 0.525) compared to the amiodarone group (Figure 3).

#### 3.1.4. The Effect of Chronic and Acute Administration of Silymarin on CaCl_2_-Induced Arrhythmia 3 Min after the Induction of Arrhythmia in Comparison with Quinidine

**Figure 4:**
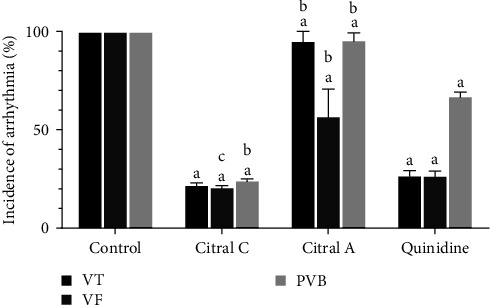
The effect of chronic (50 mg/kg i.p. administration over to 2 weeks, silymarin) and acute (100 mg/kg i.v. administration immediately after induction of arrhythmia, silymarin A) administration of silymarin on CaCl_2_-induced arrhythmia 3 min after the induction of arrhythmia in comparison with quinidine (10 mg/kg). The information in the control group was considered 100% and was compared with other groups. PVB, premature ventricular beat; VT, ventricular tachycardia; and VF, ventricular fibrillation. ^a^*P*  < 0.001 against; ^b^*P*  < 0.001 against quinidine; and ^c^*P*  < 0.05 against quinidine.

The effect of chronic and acute administration of silymarin on arrhythmia caused by CaCl_2_ was investigated 3 min after the induction of arrhythmia compared to quinidine. The incidence of VT in the chronic silymarin group had a significant decrease compared to the control group (*P* < 0.001, *d* = 0.965); it had no significant difference with other groups. The incidence of VF decreased in the chronic silymarin group compared to the control group (*P* < 0.001, *d* = 0.804) and quinidine (*P*=0.017, *d* = 0.471). The incidence of VT, VF, and PVB in the acute silymarin group had a significant decrease compared to quinidine group (*P* < 0.001, *d* = 0.957). There was a decrease in the incidence of VT and PVB in the chronic silymarin group compared to the acute silymarin group; there was a nonsignificant increase in the VF incidence (Figure 4).

### 3.2. Heart Rate before and after the Induction of Arrhythmia

**Figure 5:**
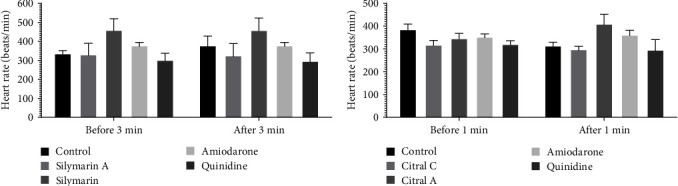
The effect of chronic (50 mg/kg i.p. administration over 2 weeks, silymarin) and acute (100 mg/kg i.v. administration immediately after induction of arrhythmia, silymarin A) administration of silymarin on heart rate before and after (a) 3 min and (b) 1 min of induction of arrhythmia.

No significant difference was observed between the heart rate measured before and after the induction of arrhythmia in different groups (Figure 5).

### 3.3. Evaluation of Antioxidant Parameters of Heart Tissue

**Figure 6:**
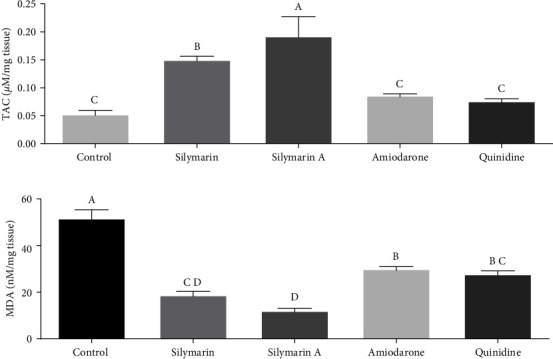
The effect of chronic (50 mg/kg i.p. administration over to 2 weeks, silymarin) and acute (100 mg/kg i.v. administration immediately after induction of arrhythmia, silymarin A) administration of silymarin on CaCl_2_-induce doxidative stress: (a) total antioxidant capacity (TAC) of the heart tissue and (b) malondialdehyde (MDA) in heart tissue. The results were expressed as mean ± SEM. Different letters indicate statistically significant differences (*P*  < 0.05).

Administration of silymarin acutely and chronically increased TAC activity compared to the control group (*P* < 0.0001, *d* = 0.929), as well as the groups treated with quinidine and amiodarone (*P* < 0.001, *d* = 0.949). In the acute silymarin group, TAC activity significantly increased compared to the silymarin group (*P*=0.019, *d* = 0.782) (Figure 6(a)). Administration of silymarin in acute and chronic forms has increased MDA level compared to the control group (*P* < 0.0001, *d* = 0.961), as well as the groups treated with quinidine and amiodarone (*P* < 0.0001, *d* = 0.831) (Figure 6(b)).

The results of the Pearson correlation showed that with the increase in TAC, the rate of VT (*P*=0.001) and VF (*P*  < 0.001) significantly decreased. On the other hand, the increase in MDA level was associated with a significant increase in the incidence of VT (*P*  < 0.001) and VF (*P*  < 0.001) (Table 1).

## 4. Discussion

We found that silymarin could reduce the incidence of VT, VF, and PVB in all groups compared to the control group. Among the groups treated with silymarin, although there was no significant difference, the best result was observed in the group which received an acute i.v. injection of silymarin at a dose of 100 mg/kg immediately after the induction of arrhythmia. Compared to other groups, all three forms of arrhythmias, especially VF, showed a greater decrease in incidence. Compared to antiarrhythmic drugs, silymarin caused a comparable reduction in the incidence of arrhythmias caused by CaCl_2_ injection. Several evidence have proven that oxidative stress plays an important role in the pathogenesis and progression of cardiovascular diseases, including heart failure and arrhythmias [30]. Atrial fibrillation and heart failure (a condition with an increased risk of arrhythmia) are associated with excess amounts of reactive oxygen species (ROS). There are several possible mechanisms for ROS to cause arrhythmias, ROS can cause focal activation and relapse. ROS alters several cardiac ion currents, promotes cardiac fibrosis, and impairs gap junction function. Activation of Ca2/CaM-dependent kinase II, tyrosine kinase c-Src, protein kinase C, and abnormal connection of cardiac sodium channels are among the recent molecular mechanisms of ROS-induced arrhythmia. Ion channel blockage has important limitations for chronic treatment and prevention of those arrhythmias. Most of the clinical risk factors of AF, such as hypertension, age, and cardiac surgeries, are disorders associated with oxidative stress [11]. Serum markers are increased in these patients [10]. Cardiomyopathy is also accompanied by oxidative stress, which significantly increases the incidence of VT/VF [12]. Increased production of reactive oxygen species plays a role in causing heart failure because oxidative stress seems to play a role in heart regeneration, mechanical isolation, and changing calcium sensitivity. Most likely, actin oxidation affects contractile performance. This concept is strongly supported by the present findings [12]. Late INa block by TTX or ranolazine reduces the harmful effects of hydrogen peroxide on electrical and contractile functions, cellular homeostasis, and cellular Ca^2+^ of cardiac myocytes. The results of the study have shown that the increase in late INa is a major ionic mechanism underlying the cardiac actions of hydrogen peroxide. Decreasing late INa may be an important step to reduce ROS-induced myocardial disorders [31]. Reactive oxygen species, an inevitable product of oxidative phosphorylation, cause protein modification, lipid peroxidation, and mitochondrial DNA (mtDNA) damage, which ultimately leads to mitochondrial dysfunction. Since mtDNA encodes several OXPHOS gene subunits, ROS-induced mutations in mtDNA cause more ROS production through impaired oxidative phosphorylation [23]. Also, H_2_O_2_ perfusion in the isolated heart has induced VT/VF and AF [32], which shows the effect of increasing ROS in causing arrhythmia. ROS can cause focal activity; ROS increases the length of the action potential and causes early and late subsequent depolarization [33]. The increase of ROS affects the flow of several ions in cardiomyocytes, its effect on sodium flow is important and can cause arrhythmia. One of the mechanisms of prolongation of the action potential is due to the increase of delayed sodium current [31]. Oxidative stress facilitates myocardial fibrosis [34]. The characteristics of the extracellular matrix are important factors that can affect the propagation of the action potential in the heart. For example, the increase in collagen deposition in the extracellular matrix following cardiac infarction may create a barrier against action potential propagation and cause reentry [35]. Some PVBs are caused by stray impulses or reentry signals and originate around the edges of the infarcted or ischemic areas of the heart [36]. Since ischemia is one of the causes of ventricular arrhythmia and the reason is the reentry signals caused by oxidative stress, here too ROS causes early and delayed depolarization. Therefore, silymarin has a reducing effect on this disease with its antioxidant effect. Research shows that silymarin has significant antioxidant properties. Antioxidant effect of silymarin in different ways, including cleaning of free radicals [21, 22] preventing the formation of free radicals by inhibiting enzymes or maintaining the electron transport chain in mitochondria under stress conditions [23], participating in maintaining the optimal state of regeneration by activating enzymatic and nonenzymatic antioxidants through transcription factors such as Nrf2 and NF-*κ*B [24, 25], activating vitagens responsible for the production of protective molecules including HSP and thioredoxin and providing more protection is created in stress conditions [26, 27], and therefore, it reduces arrhythmias. The present study showed that oxidative stress can be an upstream target in the treatment of arrhythmia. In this study, it was shown that silymarin has the ability to enhance antioxidant activity and decrease lipid peroxidation. To put it simply, silymarin helps to regulate oxidative stress in the heart by strengthening antioxidant activity.

Increased risk of ventricular arrhythmias can be associated with QT prolongation [37], which pretreatment with silymarin has reduced cardiac excitability due to doxorubicin administration [38]. In this study, silymarin decreased the QT interval. Silymarin, as an antioxidant agent, has antiplatelet and anticoagulant effects, which makes it possess a protective effect against thrombosis [14]. Silymarin has had a significant role in the prevention of ischemia–reperfusion injury of the heart. This compound has been able to reduce the damage caused by reperfusion, which is one of the important factors in causing arrhythmia and sudden cardiac death [39, 40]. Silymarin has also been shown to have antiatherosclerosis properties in rabbits with hypercholesterolemia [41]. Silymarin has hypolipidemic effects, which have been shown to reduce total cholesterol and triglycerides [42]. On the other hand, it has increased HDL [43]. In this way, it can play an important role in preventing heart diseases caused by hyperlipidemia.

Currently, amiodarone is the most widely used antiarrhythmic drug and is one of class 3 drugs. Amiodarone blocks potassium channels, sodium channels, calcium channels, and beta receptors, and increases the action potential duration and QT interval [44, 45]. Quinidine acts as a class I antiarrhythmic agent by blocking the rapid flow of sodium into the cell. This current is responsible for rapid depolarization and conduction of the action potential in the heart [1]. In the present study, silymarin was able to control arrhythmias in a way comparable to amiodarone and quinidine; the best result was observed following acute administration, the reason is probably due to the low bioavailability of silymarin and its low solubility in water which reduces its absorption in the intestine. Some classes 1 and 3 antiarrhythmic drugs, such as quinidine, may worsen existing arrhythmias [1]. Paradoxically, a common side effect of these drugs is arrhythmia [45]. In addition, amiodarone has important complications such as pulmonary fibrosis, deposition of fine crystals in the cornea, tremors, and thyroid dysfunction [45].

## 5. Conclusions

Silymarin has a preventive effect against cardiac arrhythmias and with its strong antioxidant effect, it can prevent the development of VT, VF, and PVB. Its effects worked at the same level as amiodarone and quinidine, and it was even better than these two drugs in controlling arrhythmia. In addition, the effect of acute injection of silymarin was greater than its chronic administration, and its effects were greater in the first minute of induction of arrhythmia than in the third minute. These effects probably occurred as a result of increased TAC and decreased lipid peroxidation (MDA).

## Figures and Tables

**Table 1:** Correlation between arrhythmia, total antioxidant capacity (TAC), and malondialdehyde (MDA).

	VT	VF	PVB
TAC			
Pearson correlation	−0.677 ^*∗∗*^	−0.827 ^*∗∗*^	−0.017
Significance	0.001	<0.001	0.945
*N*	20	20	20
MDA			
Pearson correlation	0.911 ^*∗∗*^	0.915 ^*∗∗*^	0.45
Significance	0.000	<0.001	0.47
*N*	20	20	20

^ _ ^*∗∗*^_^Correlation is significant at the 0.01 level. VT, ventricular tachycardia; VF, ventricular fibrillation; and PVB, premature ventricular beat.

## Data Availability

The data that support the findings of this study are not openly available due to reasons of sensitivity and are available from the corresponding author upon reasonable request.
